# Late Trial for Alleged Poisoning with Arsenic

**Published:** 1849-03

**Authors:** 


To the Editor of the Boston Medical and Surgical Journal.

*Late Trial for Alleged Poisoning with Arsenic.*

Dear Sir:—The following brief synopsis of a late trial at Worcester, for murder alleged to have been committed nearly *fifteen years ago,* was prepared, at your request, while attending as a witness for the Government.

John Cook, Jr., formerly of Ashburnham, now of Winchenden, was brought to trial Dec, 7th, 1848, in the Supreme Judicial Court, on an indictment charging the murder of his wife Roxanna in February, 1834, by administering arsenic. Chief Justice Shaw and Associate Justices Dewey and Metcalf, on the Bench. Hon. Ezra Wilkinson, of Dedham, Attorney for the Commonwealth. Hon. Benjamin F. Thomas, of Worcester, and Milton Whitney, Esq., of Fitchburg, counsel for the prisoner.

On the part of the Government, evidence was introduced to show that Cook was twelve years younger than his wife; that they were married two years previous to her death; that she had considerable property, which Cook unsuccessfully strove to get possession of. Early in February, 1834, Cook bought arsenic of Dr. Pierce in Ashburnham. Mrs. Cook was a woman of good health, and had an infant child two months old. On Sunday, February 23d, 1834, about 4 o'clock, P. M., Cook drew some cider and urged his wife to drink, telling her it had kept very sweet. In a few minutes after drinking the cider, she had severe pain at the stomach, and made ineffectual attempts to vomit. Pain and sickness became violent and paroxysmal. During the evening Cook gave his wife hot sling, which aggravated her distressing symptoms. He repeatedly refused to go for a physician, till nearly morning, when Dr. Wm. H. Cutler was called. He found her lying on a bed in great distress at the stomach, the pulse small, the extremities cold, the bowels much swollen—and noticed frequent ineffectual attempts to vomit. She was nearly comatose, and never spoke after he saw her. An emetic of ipecac. and sulph. zinc and purgative enema failed to produce either vomiting or dejections. She died at 10 o'clock, A. M., on Monday, 18 hours after drinking the cider, at which time she was in perfect health. Cook refused to have a *post-mortem* examination; would furnish nothing but his wife's *wedding dress for a shroud.* He was away from home when his wife died. When informed of her death, he made no reply. He was then walking towards his own house, in company with a girl eleven years younger than himself, whom he afterwards married. Soon after the death of his wife, Cook secured the money concerning which there had previously been some altercation.

The body of Mrs. C. was placed in a tomb. For several years afterwards it was observed, by the sexton and others, that the body continued in an unusual state of preservation. Ten years after death, the sexton, a very intelligent man, testified he could have identified the body by the *features alone*, while other bodies, entered afterwards, were totally decayed. In October, 1849, Mrs. Cook's coffin was again opened, and the chest retained its rotundity, the integuments being entire, the face and limbs being then decayed. The fact that Mrs. Cook's body exhibited such apparent tokens of preservation in the regions of the stomach and chest, together with the fact that many bodies whose sepulture in the same tomb at dates much more recent, had become totally disintegrated, served to revive and heighten suspicions which existed against Cook at the time of her death. In February, 1848, by request of the relatives, the remains of Mrs. Cook were obtained by my partner, Dr. Leonard French, and Mr. Wm. L. Lincoln, a medical student, and by myself, delivered to Prof. Webster, at Cambridge. These remains consisted of all the matter of the stomach and intestines; as whatever now remained in that portion of the coffin occupied by the abdominal cavity, its walls and contents, was taken up with a spoon and deposited in glass jars, which were closed and sealed. There was no reason to suspect that this matter had been in contact with the earth, or any source from which arsenic or other poison could have been derived. There was no plate on the coffin. The sexton was present and identified the body. All the manipulations in obtaining, securing, and conveying the remains to Prof. Webster, were conducted with the greatest care, and without the possibility of mistake. From these remains, Dr. Webster testified that he obtained, by chemical analysis, a little more than four grains of oxide of arsenic (white arsenic); which is equal to 3 and 16-000 grains of metallic arsenic. A portion of this arsenic was exhibited at the trial.

A detailed report of Prof. Webster's, analysis of this interesting case has been published, from which I transcribe the following:—

"The sealed jars with their contents were delivered to me on the evening of the 14th of February last by two medical gentlemen, one of whom had attended to the collection and removal of the remains. The seals were unbroken. They were taken to the laboratory of the Medical College and there opened, when a faint disagreeable odor was perceptible, but far less offensive than had been anticipated. The contents of both jars were turned out into a large wedgwood evaporating dish. They were dark, with different shades of brown and black; the whole were moist, but broken up into many small lumps, some of which had considerable firmness and consistency. Portions of membranes were enveloped in the mass, one of which was apparently part of the stomach or intestines, but so much changed that it was impossible to determine which.

It being improbable that any vegetable or organic poison was present, I proceeded to examine separate portions with a lens, but no particles of arsenic or other mineral substance could be detected. Of the mineral substance usually had recourse to as poisons, none appeared more likely to be found, if any such had been administered, than arsenic, or one of the oxides of that metal, and it seems pretty well settled that the oxide, known as arsenious acid, or ratsbane, resists decomposition more effectually, and for a longer time, than any other mineral poison commonly used. In recent cases of poisoning with this substance, I have more than once been able to detach minute portions from the inner surface of the stomach, particularly in another case upon which I had been engaged a few weeks before this time. It may not be superfluous to state, as showing in some degree how frequently arsenic is employed, or suspected to have been the cause of death, that in the course of the preceding three months no fewer than four cases were submitted to my examination, in two of which arsenic was found.

As it was impossible to obtain any satisfactory results by acting upon the mass of matter from the jars without previous solution, and as it was deemed important not to limit the examination to the whole solution at once, but to subject the matter to all the chemical processes which were calculated to elicit and confirm the truth, the mass was divided into separate portions.

One portion was boiled in pure distilled water for nearly an hour, and strained; the animal matter was coagulated by chlorine, which was subsequently expelled, and the clear solution was treated with hydro-sulphuric acid. A part of this solution was tested by the usual methods, as with nitrate of silver and ammonia, sulphate of copper and ammonia, lime water, &c., in presence of the gentlemen attending at that time the medical lectures in Boston. With these tests, and also with hydro-sulphuric acid, the presence of arsenic was distinctly indicated. No reliance, however, was placed on these preliminary trials, beyond the mere indication that arsenic was probably present.

Another portion of the remains was treated with nitric acid and nitrate of silver, and similar indications resulted.

Having obtained these indications of what was present in the remains, it was necessary to separate the substance, and in such a state that both its physical and chemical characters might be made out beyond question. For this purpose, and as the quantity of poison remaining was not supposed to be large, one third part of the remains left after the preliminary trials, was subjected to the action of sulphuric acid, and completely carbonized, or converted into charcoal; the purity of the acid, and especially its freedom from arsenic, having been previously ascertained.

Another third was heated with hydrochloric acid and chlorate of potassa, filtered and reduced by careful evaporation. That any arsenic acid present might be brought to the state of arsenious acid, the liquid was treated with sulphurous acid.

The remaining third part was treated with pure nitric acid, as employed by Orfila in the case of Cumon.

From these three portions, as many distinct, clear solutions were obtained, each of which was separately again tested, and each gave indications of arsenic.

Each of these solutions was again divided into three parts, and each part was subjected to the three following processes.

One portion of each was examined by the apparatus and process known as Marsh's. In this, hydrogen gas is obtained from zinc, sulphuric acid and the suspected solution. That all doubt as to the purity of the zinc and acid might be removed, and especially their freedom from arsenic, and also that of the apparatus, pure distilled water was first used, The hydrogen was evolved and continued a sufficient length of time to satisfy me that no arsenic was present. Had there been even a minute quantity it would have been taken up by the nascent hydrogen, and have caused a peculiar change in the appearance of the gas while burning, and other effects sufficient to detect impurity.

The apparatus, after thorough cleaning, was now charged with a fresh piece of the zinc, acid and solution, from the remains; the hydrogen evolved was ignited, and the bluish tint of the flame at once indicated the presence of arsenic. The flame was then brought in contact with a white surface of porcelain, and dark spots were produced; the same took place when a piece of clean window glass was held over the flame. Aware that other metals, if present, might produce similar spots, especially if the gas was rapidly evolved, the process was allowed to go on very slowly and continued nearly an hour.

The spots were tested and were arsenical.

Another portion of the solution was now used with the same precautions as before, and the gas was transmitted through a fine tube of glass, containing no arsenic or lead in its composition, and capable of bearing a high degree of heat. As soon as the hydrogen had displaced the air, and was issuing from the open end of the tube, a spirit lamp was placed beneath its central part and that was heated to redness. The decomposition of the gas took place, and a metallic looking crust was formed within the tube, a little distant beyond the red hot portion. No deposit was observed in the hot part of the tube, which would have occurred had any other metal than arsenic been present. The process was kept up as slowly as possible, and continued over two hours. The metallic deposit was then carefully collected and tested in various ways, so as to leave no doubt as to its nature,—it was arsenic.

The process of Marsh has not proved as infallible as was anticipated when it was first announced, and it was found that antimony, for instance, which may have been administered to a patient in some one of its forms, would give a compound gas with hydrogen that burns with a flame, the color of which is so similar to that of arseniuretted hydrogen, the two might readily be confounded; and, moreover, that a similar stain would be produced by antimony upon the porcelain. Fortunately it was discovered that all doubt could be removed by the very simple modification of the apparatus and process above described. The arsenic, if present, is volatilized in passing along the hot part of the little tube, and condenses in the cool part, but antimony fuses and retains much lustre.

The tube was now detached from the apparatus and the metallic crust was heated; brilliant points began to appear in the upper part, which increased in size and number until distinct octohedral crystals with high lustre could be distinguished by the naked eye. There could be little, if any doubt, that they were crystals of arsenious acid.

To leave no doubt on this point, the crystals were dissolved out by boiling water, and the solution again tested; the result confirmed the previous one.

Another portion of the solution from the remains was treated with hydrosulphuric acid gas; a copious yellow precipitate was obtained, also indicating the presence of arsenic. But although much reliance has been placed upon this test, it is by no means conclusive; for several substances may be present which will give a yellow color when there is no arsenic. The yellow sulphuret of arsenic may, however, be distinguished by reduction, and if arsenic is present, it is easily separated and obtained in the metallic state, in a small tube, and can then be converted into arsenious acid, and crystallized as before described. This was done, and the result confirmed the other experiments.

Another process to which portions of the suspected solution were submitted, consisted of boiling slips of bright copper in the liquid, with a small quantity of hydrochloric acid, known as Reinsch's process. This process is not so delicate as Marsh's, but gives very satisfactory results, with proper precaution, and was resorted to as confirmatory of the other experiments. By this, the arsenic is deposited upon the copper, and can be removed and subjected to the other tests. As to the quantity that can be detected by Reinsch's process, Dr. Christison, high authority, tells us it will detect "at least a 250,000th part of arsenic in solution." Others say it will detect a 3000th of a grain in 90,000 times its weight in water. But the production of a mere stain upon the copper is not decisive. The copper may be thus tarnished when no arsenic is present; it may be occasioned by organic matters. It is not a process to be solely relied upon for medico-legal purposes. The production of the metallic crusts, and their conversion into arsenious acid and reconversion to metallic arsenic, in each case fully tested in other ways, can alone satisfy the operator.

The most complete method of distinguishing arsenic from antimony, is by converting the arsenical stain into chloride of arsenic, by exposure to chlorine; the chloride may then be converted into the pale yellow sulphuret, which, with ammonia, affords a colorless solution;—by heat the ammonia is volatilized, and the yellow sulphuret is reproduced. The action of nitric and hydrochloric acids, and subsequent evaporation to dryness, afford white rings of arsenic acid, which deliquesce and become invisible; and the deliquesced acid gives, with nitrate of silver, the dirty red stain of arseniate of silver. According to M. Divergie, this series of actions is conclusive. But we have no statement how small a quantity of arsenic or antimony may, in this way, be distinguished.

As the fact to be ascertained in the case of Cook was of such vast importance, and no case, as far as I have discovered, is on record where arsenic had been detected after the the lapse of so many years, it was deemed imperative on me to subject the solutions obtained from the remains, to every form and method of examination which could throw light upon it; hence, although I have used the terms *tube*, *solution*, &c., it has been done for the sake of brevity, but in fact several distinct portions of the liquid and several tubes were employed—and the fact that arsenic was present in the remains, I consider fully established. How it came to be there, I was not called upon to inquire.

In regard to the question, always, I believe, asked in judicial proceedings, as to the antiseptic power of arsenic, deemed of so much importance in the present case, it does not appear that the popular notion is fully sustained. Experiments have been made by Iager, on the bodies of animals poisoned by arsenic, which go to show that it neither retards nor hastens putrefaction. Seeman's experiments upon dogs show that their bodies putrified as usual. It is probable that the antiseptic power of arsenic has been much over-estimated.

Still there can be no doubt that arsenious acid combines with the fatty and albuminous tissues, and forms compounds which are not susceptible of alteration under ordinary circumstances; and some instances have occurred where the bodies of persons poisoned by arsenic have been found, long after death, in a remarkable state of preservation. In some of these the stomach and intestines have been preserved, although the general mass of the body had disappeared, leading to the detection, of and bringing to light crimes committed many years before.

Another question is probably always put to a witness in a case of suspected poisoning by arsenic, viz: What is a fatal dose? So many and various are the circumstances and conditions, that it is doubtful if any definite answer can be given. We have the case of Bertrand, who took five grains on an empty stomach, with no ill effects—and we have high authority that four and a half grains is a fatal dose. In a case in New York, an ounce was taken, and the pain in the stomach was but slight. Then, again, arsenic does not always produce violent and well-marked symptoms, altho' fatal. It may kill, too, by its narcotic operation, no morbid condition of the stomach being observed after death.

As to the existence of arsenic in the bones, normal arsenic, the early opinion of Orfila, that such is the fact, has not been confirmed by later researches. If found in the state of arseniate of lime, as was affirmed by Couerbe, this would not affect the question as to its presence in the stomach and intestines. In the case of Cook, no portion of the bones was examined.

But Orfila has retracted his former opinion, and a few years ago forwarded to the Academy of Medicine of Paris a statement that he could no longer extract arsenic from bones."

A very large number of medical witnesses were called for the government, and also for the defence. No exceptions were taken to Dr. Webster's chemical evidence, and no attempt was made to discredit the results of his analysis. There was a concurrence of opinion with the physicians that the symptoms of which Mrs. Cook died were consistent with poisoning by arsenic, although vomiting and purging were absent; and also in favor of the antiseptic power of that poison. For the defence, evidence was introduced that contradicted and in some degree impeached one of the principal government witnesses. The chief Justice charged the jury favorably for the prisoner. After deliberating forty minutes, the jury returned a verdict of NOT GUILTY.

ASHBY, Dec., 1848. ALFRED HITCHCOCK.

